# Diagnostic Accuracy of Neurocognitive and Executive Functions to Discriminate Women With and Without Fibromyalgia Syndrome: A Secondary Analysis

**DOI:** 10.3390/jcm13206195

**Published:** 2024-10-17

**Authors:** Margarita Cigarán-Mendez, Juan C. Pacho-Hernández, Ángela Tejera-Alonso, Cristina Gómez-Calero, César Fernández-de-las-Peñas, Juan A. Valera-Calero, Francisco G. Fernández-Palacios

**Affiliations:** 1 Department of Psychology, Universidad Rey Juan Carlos, 28922 Alcorcón, Spain; margarita.cigaran@urjc.es (M.C.-M.); angela.tejera@urjc.es (Á.T.-A.); gines.fernandez@urjc.es (F.G.F.-P.); 2 Escuela Internacional de Doctorado, Universidad Rey Juan Carlos, 28922 Alcorcón, Spain; 3 Department Physical Therapy, Occupational Therapy, Rehabilitation, and Physical Medicine, Universidad Rey Juan Carlos, 28922 Alcorcón, Spain; cristina.gomez@urjc.es (C.G.-C.); cesar.fernandez@urjc.es (C.F.-d.-l.-P.); 4 Department of Radiology, Rehabilitation and Physiotherapy, Faculty of Nursery, Physiotherapy and Podiatry, Complutense University of Madrid, 28040 Madrid, Spain; juavaler@ucm.es; 5 Grupo InPhysio, Instituto de Investigación Sanitaria del Hospital Clínico San Carlos (IdISSC), 28040 Madrid, Spain

**Keywords:** fibromyalgia, diagnostic accuracy, mental inhibition, cognitive, execution

## Abstract

**Objective:** The aim of the current study was to determine the capability of neurocognitive variables and executive functions to differentiate women with and without fibromyalgia syndrome (FMS). **Methods:** A secondary diagnostic accuracy analysis was conducted. A battery of neurocognitive and executive function tests (the D2 Attention test, the Rey-Osterrieth Complex Figure for visual perception, “Digits D/R/I” tests of the WAIS-IV battery for working memory, the 5-Digit test for mental inhibition, the Symbol Search for processing speed, and the Zoo Test for planning/decision-making) were collected in 129 women with FMS and 111 without FMS. The area under the receiver operating characteristic (ROC) curve, optimal cut-off point, sensitivity, specificity, and positive and negative likelihood ratios (LR) for each variable were calculated. **Results:** Between-group differences were identified in ROCF_Copy (*p* = 0.043), ROCF_Recall (*p* = 0.004), d2_TR (*p* = 0.019), d2_TA (*p* = 0.007), d2_TOT (*p* = 0.005), d2_CON (*p* = 0.004), d2_C (*p* = 0.042), Symbol Search (*p* = 0.008), Decoding _FDT (*p* = 0.001), Retrieving_FDT (*p* = 0.001), and Inhibiting_FDT (*p* = 0.024). The result showed that FDT-based outcomes (Retrieving_FDT: ROC 0.739, sensitivity 85.3%, specificity 48.6%; Decoding_FDT: ROC 0.724, sensitivity 50.4%, specificity 16.2%; Inhibiting_FDT: ROC 0.708, sensitivity 56.6%, specificity 22.5%) were the variables able to differentiate between women with and without FMS. **Conclusions:** Although women with FMS exhibited deficits in attention, long-term visual memory, processing speed, and mental inhibition when compared with women without FMS, only mental inhibition scores showed moderate diagnostic accuracy to discriminate between women with and without FMS. Future studies investigating these results in clinical settings are needed to identify the clinical relevance of these findings.

## 1. Introduction

Fibromyalgia syndrome (FMS) is a multidimensional pain condition showing a worldwide prevalence of 6% [1]. Thus, FMS represents an important economic burden to health care systems, since the annual direct cost/patient can range from $ 1750 to $ 35,920 in the United States of America and from $ 1250 to $ 8504 in European countries [2]. Individuals with FMS exhibit a plethora of physical (i.e., generalized pain, fatigue, muscle weakness), psychological (i.e., anxiety, depression) and cognitive (i.e., memory loss, attention deficit) symptoms [3,4]. All of these symptoms seem to be interconnected into complex associations [5] leading to a decreased health-related quality of life [6].

Although widespread pain and fatigue are probably the most self-reported symptoms experienced by individuals with FMS, the presence of cognitive impairments, also known as “fibro fog” has received particular attention in the last decades [7]. Fibro fog is defined as those cognitive problems (e.g., memory problems, attention deficits, orientation, and general confusion) self-described by patients with FMS during daily life activities [8]. Current evidence suggests that cognitive impairments in FMS are heterogeneous and seem to be domain-specific [9,10]. For instance, Wu et al. [9] concluded that differences in learning/memory and attention/psychomotor speed between women with and without FMS were significant (large pooled clinical effect) whereas differences in executive functions and working memory were smaller (medium pooled clinical effect) when comparing women with and without FMS. The meta-analysis by Bell et al. [10] identified differences (moderate to large clinical effect) in other domains e.g., deficits in inhibitory control, problems with short- and long-term memory, and task switching between women with and without FMS.

These discrepancies can be explained by the fact that psychological and emotional factors could differently affect neurocognitive domains [11,12]. Accordingly, studies investigating differences in specific cognitive and executive performance domains considering these potential cofounder factors in patients with FMS are needed. Our research group has recently investigated differences in neurocognitive and executive functions between women with and without FMS by controlling those factors that could potentially influence these domains, such as depression, anxiety, sleep quality, and hypervigilance [13]. We found that women with FMS exhibited deficits in some domains such as selective attention, long-term visual memory, processing speed, and mental inhibition, but not in others including working memory or planning, when compared with asymptomatic women. Thus, these deficits were not affected by anxiety levels, depressive symptoms, poor sleep quality, or pain hypervigilance in our sample [13]. Most published studies have investigated differences between individuals with (patients) and without (controls) FMS [9,10,11,12,13]. No study has previously investigated whether these differences in neurocognitive and/or executive functions are able to help in the diagnosis of individuals with FMS. Therefore, the aim of this study was to determine the capability of neurocognitive variables and executive functions to differentiate women with and without FMS by analyzing the area under the receiver operating characteristic (ROC) curve, the optimal cut-off point, and the sensitivity, specificity, and positive and negative likelihood ratio (LR) for each variable.

## 2. Methods

### 2.1. Participants

We present here a secondary analysis from our previously published study [13]. As previously described [13], this study included women diagnosed with FMS by their rheumatologists according to the diagnostic criteria of the American College of Rheumatism (ACR) as modified in 2010 [14]. These women were recruited from various Fibromyalgia Associations in Madrid (Spain). Further, women without a history of chronic pain were recruited through local social media advertisements. The exclusion criteria were: (1) prior whiplash injury; (2) previous surgeries; (3) other underlying medical conditions; (4) neuropathic pain, e.g., radiculopathy; (5) a current psychiatric diagnosis based on DSM-V (e.g., major or mild neurocognitive disorders, schizophrenia); or (6) taking medications, e.g., antipsychotics, anticonvulsants, or anticholinergics) that affect cognition [13].

The study was approved by the Local Ethics Committee of the Universidad Rey Juan Carlos (URJC 2508202218222), and all participants provided written informed consent before their participation.

### 2.2. Study Procedure

Participants attended a single session lasting 90 min at the experimental laboratory of the Universidad Rey Juan Carlos (Madrid, Spain) between May 2022 and May 2023. All sessions were conducted by experienced clinical neuropsychologists with more than ten years of experience. Each session was individualized and included the following tests covering different neurocognitive and executive functions (Table 1).

### 2.3. Visuospatial Memory

Visual perception, visuo-construction ability, and spontaneous memory retention were assessed using the Rey-Osterrieth Complex Figure (ROCF) test [15]. This test evaluates the participant’s ability to remember visual details, to organize and integrate parts of a figure, and mentally manipulate the figure. Participants are first asked to copy a geometric figure (composed of 18 black lines) onto a sheet of paper. They are then asked to reproduce the figure from memory immediately (immediate recall) and after 20–30 min (delayed recall), without the original figure present. No instructions are provided to memorize the figure, as the task aims to measure what is spontaneously retained. The test provides several scores: ROCF_Copy, immediate recall, and delayed recall points (calculated by dividing copy points by maximum points); ROCF_Recall, which is the percentage of the recall (delayed recall points divided by immediate recall points); and ROCF_TimeCopy, the time taken to copy the figure. The ROCF has demonstrated good psychometric properties [16] and excellent intra- and inter-rater reliability (ICC ranging from 0.85 to 0.97) for the total score [17].

### 2.4. Selective Attention

Selective attention and concentration were measured using the Spanish version of the d2 Attention test (d2) [18]. Selective attention refers to the ability to focus on relevant aspects of a task while ignoring irrelevant ones, doing both quickly and accurately [19]. The d2 test consists of 14 lines, each containing 47 characters, totaling 658 items. Participants must scan each line from left to right and mark every letter “d” with two small dashes (either both above, both below, or one above and one below). These are the target items, while other combinations, such as “p” or “d” with one or no dashes, are irrelevant. Participants have 20 s to complete each line, and the entire test takes 8 to 10 min. The test provides several scores: d2_TR (total items attempted across the 14 lines), d2_TA (correctly identified relevant items), d2_O (relevant items missed and/or omitted), d2_C (irrelevant items mistakenly marked), d2_TOT (total test effectiveness, calculated as TR − (O + C)), d2_CON (concentration index, calculated as TA − C), d2_TR+ (the line with the most attempted items), d2_TR- (the line with the fewest attempted items), and d2_VAR (variation index, calculated as the difference between TR+ and TR−). The d2 test has shown good construct validity and good to excellent test-retest reliability (ICC ranging from 0.78 to 0.94) [20].

### 2.5. Executive Functions

Processing speed was measured using the “Symbol Search” (SS) subtest of the Wechsler Adult Intelligence Scale (WAIS-IV) battery [21]. This is a paper-and-pencil test comprising two sections: a key area with nine nonsensical pairs of digits and symbols, and a response area where digits are randomly placed alongside blank spaces. Participants are required to fill in the blanks with the corresponding symbols from the key as quickly as possible within 120 s. The SS has shown internal construct [22] and good test-retest reliability with coefficients ranging from 0.70 to 0.80) [23].

Working memory was assessed using the “Digits D/R/I” subtest of the WAIS-IV battery [24]. This test consists of three tasks: digit span forward (DSF), where participants are asked to repeat a series of orally presented digits in the same order they are given; digit span backward (DSB), where participants are required to repeat the digits in reverse order; and digit span sequencing (DSS), where participants must repeat the digits read by the examiner in ascending numerical order. Digit Span tests have shown good construct validity for assessing working memory [25].

Mental inhibition was evaluated through the “response inhibition index” of the 5-Digit Test (FDT) [26] which is a Stroop-like task consisting of four parts: Reading, Counting, Election, and Alternation. Each part contains 50 items. The Reading and Counting tasks measure automatic and simple cognitive processes, while the Election and Alternation tasks assess more complex processes. The score is calculated by multiplying the number of errors made by the time taken to complete each part. The resulting scores include: Decoding_FDT, which represents the time (in sec) needed to read all numeric items; Retrieving_FDT, which reflects the time (in seconds) taken to read all non-numeric items (e.g., asterisks); Inhibiting_FDT, the time needed to read the same numeric item repeatedly; and Shifting_FDT, the time required to read a set of mixed numeric items in a box. The FDT exhibited moderate-to-excellent test-retest reliability (ICC from 0.59 to 0.97) [27].

Planning and decision-making were evaluated using the Zoo Map Test [28], which assesses the ability to organize, plan, and solve problems to achieve a specific goal. This test consists of two parts: the first part assesses planning ability in a context where no predefined pattern is required and everything depends on the individual’s own decisions, while the second part evaluates the individual ability to apply a concrete external strategy. For each version, errors made are subtracted from the sequence score on the test sheet. The scores from both parts are then added together to generate a total sequence-error score, ranging from 0 to 16 points. A score between 11 and 16 is considered within the normal range, whereas a score of 10 or lower indicates some degree of deficiency. Oosterman et al. [29] found that the Zoo Map Test is a valid indicator of planning and decision-making.

### 2.6. Sample Size Determination

The sample size for the current study was calculated based on the diagnostic accuracy analysis planned. Since the study aimed to detect neurocognitive differences between women with and without FMS using ROC curve analysis, we followed the recommendations outlined by Bujang et al. [30]. A minimum sample size of 100 per group is recommended for a study aimed at detecting a moderate effect size (AUC ≥ 0.7) with adequate power (≥80%) and a significance level of 5% [30].

### 2.7. Statistical Analysis

Data processing and analyses were performed using the Statistical Package for the Social Sciences (SPSS) v.29.1.1 (Armonk, NY, USA) for Mac OS. All tests were two-tailed, with a significance threshold set at *p* < 0.05. Initially, histograms and Shapiro-Wilk tests were used to evaluate the distribution of continuous variables. Descriptive statistics were displayed to outline the sample’s demographic and neurocognitive characteristics.

The differences in neurocognitive variables and executive functions between women with and without FMS were analyzed using independent samples in Student’s *t*-tests.

A diagnostic accuracy analysis was conducted to determine the ability of neurocognitive variables and executive functions to distinguish women with FMS from asymptomatic pain-free women. This analysis was conducted using the area under the receiver operating characteristic (ROC) curve, where ROC values ≥ 0.7 indicate acceptable discrimination [31]. The optimal cut-off point for each variable was determined using the Youden index [32], with metrics such as sensitivity, specificity, positive likelihood ratio (LR), and negative LR reported. The validity was considered acceptable if the sensitivity was at least 70% and specificity at least 50% [33].

## 3. Results

As previously reported [13], from a sample of 150 women with FMS screened against the eligibility criteria and 125 asymptomatic pain-free women who responded to different announcements, 129 women with FMS (age: 54.7, SD: 9.4 years) and 111 asymptomatic women (mean age: 55.3; SD: 13.9 years) were included. Between-group comparisons of neurocognitive variables and executive functions can be observed in Table 2. Significant between-group differences were identified for ROCF_Copy (*p* = 0.043), ROCF_Recall (*p* = 0.004), d2_TR (*p* = 0.019), d2_TA (*p* = 0.007), d2_TOT (*p* = 0.005), d2_CON (*p* = 0.004), d2_C (*p* = 0.042), Symbol Search (*p* = 0.008), Decoding _FDT (*p* = 0.001), Retrieving_FDT (*p* = 0.001) and Inhibiting_FDT (*p* = 0.024) [13].

Table 3 shows the diagnostic accuracy of all of the neurocognitive variables assessed in differentiating women with and without FMS, as assessed using ROC curve analysis. The ROC values indicate how well each test can discriminate between groups, with values closer to 1.0 representing better discrimination. As shown in Table 3, just three variables showed acceptable ROC values (≥0.7). The highest ROC value was observed for Retrieving_FDT (0.739), which was indicative of a good discriminatory power. This test also showed high sensitivity (85.3%), meaning that it accurately identified most FMS women (positive LR 1.660), although its specificity was moderate (48.6%), suggesting that it was less effective for excluding women without FMS (negative LR 0.302). Similarly, Decoding_FDT exhibited an ROC of 0.724, with moderate sensitivity (50.4%) and low specificity (16.2%). Despite this, its high ROC indicates that this score has strong overall discriminatory ability. Thus, Inhibiting_FDT, with an ROC of 0.708, also had acceptable discriminatory ability; however, it has lower sensitivity (56.6%) and specificity (22.5%) compared to Retrieving_FDT, suggesting it could be less useful in clinical settings. These results collectively revealed that FDT-based outcomes were the most effective variables for differentiating between women with and without FMS.

In contrast, the remaining neurocognitive variables exhibited ROC values below the acceptable threshold of 0.7, indicating poor diagnostic performance. Variables such as ROCF_Copy (0.457), ROCF_Recall (0.391), and d2_TA (0.323) exhibited low ROC values and showed no significant ability to discriminate between women with and without FMS. Many of these variables, including ROCF_Recall and d2_TA, have zero sensitivity and specificity, with corresponding Youden indices of 0.000, suggesting they do not offer any meaningful diagnostic value. Similarly, other variables like Zoo Map Test (0.401) and Symbol Search (0.341) also fall short of the threshold, indicating limited diagnostic utility. While certain tests, such as DSF and DSS, showed perfect sensitivity (100%) and very high specificity (99.1%), their ROC values (both around 0.396) suggest that, despite their high likelihood ratios, they do not perform well overall in distinguishing between women with and without FMS.

**Figure 1:**
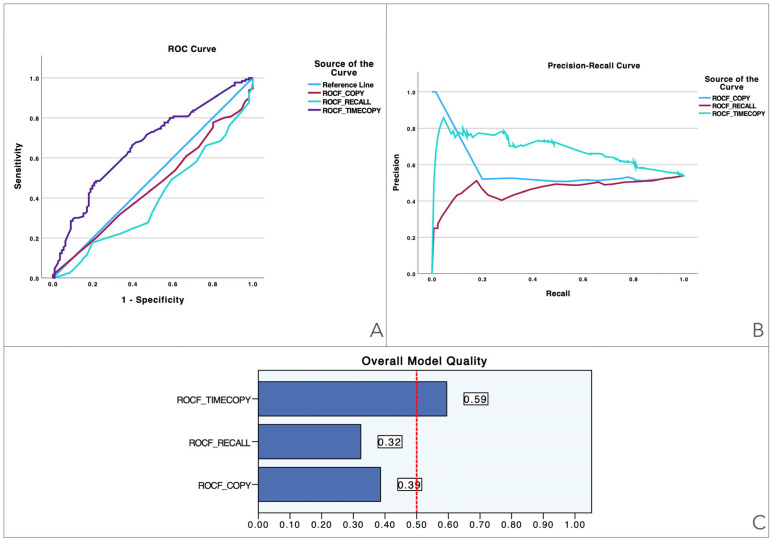
Visualization of model quality by using ROC curves and Precision-Recall curves for each domain of the Rey-Osterrieth Complex Figure (ROCF). The ROC curve (**A**) shows the trade-off between sensitivity and specificity for each domain while the Precision-Recall curve (**B**) further details the performance of these domains. The bar charts (**C**) quantify the overall model quality for each domain.

**Figure 2:**
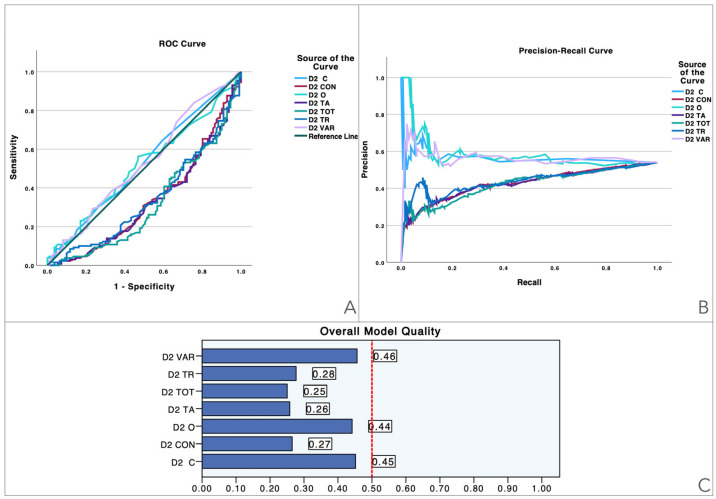
Visualization of model quality by using ROC curves and Precision-Recall curves for each domain of the d2 attention test. The ROC curve (**A**) shows the trade-off between sensitivity and specificity for each domain while the Precision-Recall curve (**B**) further details the performance of these domains. The bar charts (**C**) quantify the overall model quality for each domain.

**Figure 3:**
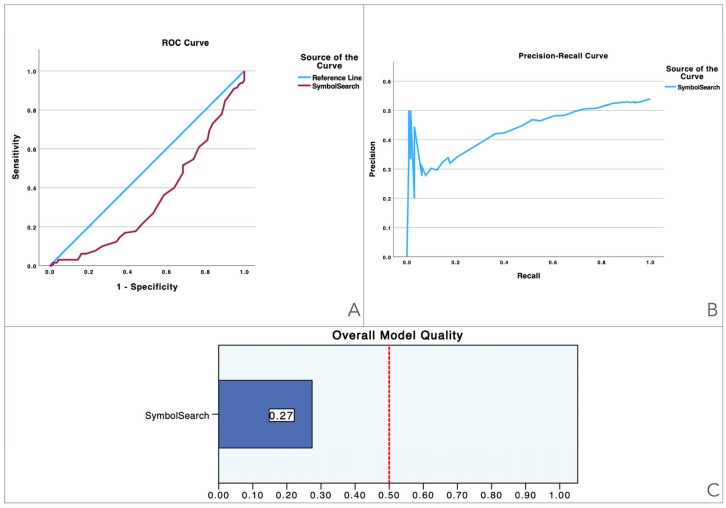
Visualization of model quality by using ROC curves and Precision-Recall curves for the Symbol Search. The ROC curve (**A**) shows the trade-off between sensitivity and specificity while the Precision-Recall curve (**B**) further details the performance of the variable. The bar charts (**C**) quantify the overall model quality.

**Figure 4:**
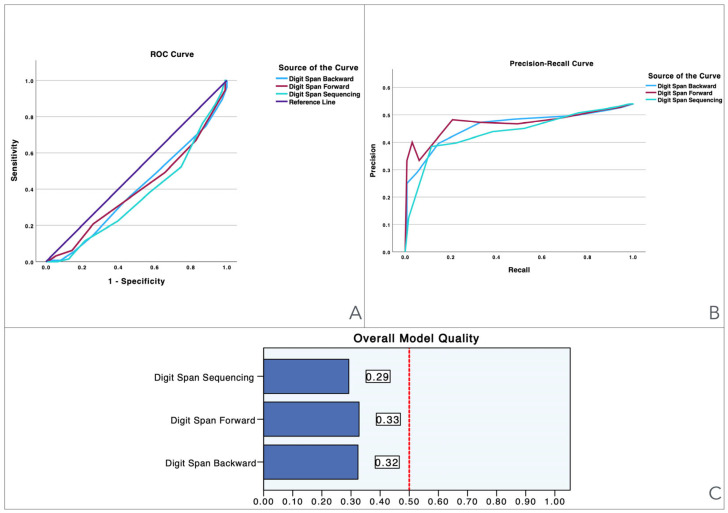
Visualization of model quality by using ROC curves and Precision-Recall curves for each domain of Digits D/R/I test. The ROC curve (**A**) shows the trade-off between sensitivity and specificity for each domain while the Precision-Recall curve (**B**) further details the performance of these domains. The bar charts (**C**) quantify the overall model quality for each domain.

**Figure 5:**
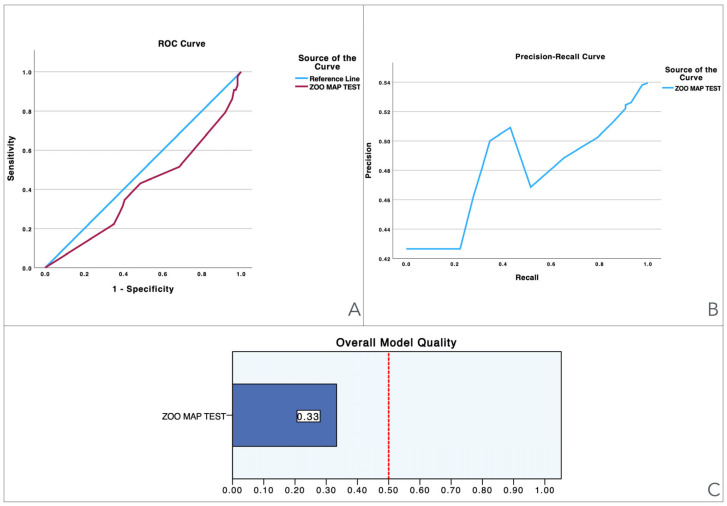
Visualization of model quality by using ROC curves and Precision-Recall curves for the Zoo Map Test. The ROC curve (**A**) shows the trade-off between sensitivity and specificity while the Precision-Recall curve (**B**) further details the performance of the variable. The bar charts (**C**) quantify the overall model quality.

**Figure 6:**
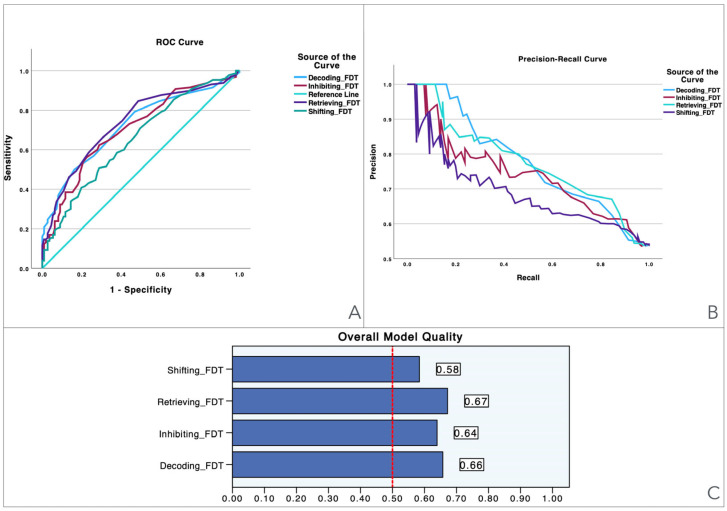
Visualization of model quality by using ROC curves and Precision-Recall curves for each domain of the Five Digit Test (FDT). The ROC curve (**A**) shows the trade-off between sensitivity and specificity for each domain while the Precision-Recall curve (**B**) further details the performance of these domains. The bar charts (**C**) quantify the overall model quality for each domain.

To provide a visual illustration of these findings, Figure 1, Figure 2, Figure 3, Figure 4, Figure 5 and Figure 6 illustrate the discriminant capacity of each domain: visuospatial memory (ROCF, Figure 1), selective attention (d2 test, Figure 2), processing speed (Symbol Search, Figure 3), working memory (Digits D/R/I, Figure 4), planning or decision (Zoo Map, Figure 5), and mental inhibition (Five Digit Test, Figure 6) by providing their ROC curves, Precision-Recall curves, and a summary of the overall model quality. As can be observed, mental inhibition, as evaluated by FDT-based outcomes, stands out with the highest AUC, indicating superior discriminatory power compared to the remaining neurocognitive variables and executive functions.

The Precision-Recall curves plot precision (the ratio of true positives to all predicted positives) against recall (the ratio of true positives to all actual positives) across different threshold settings. They are particularly useful for evaluating models based on imbalanced datasets, where one class is much rarer than the other. Unlike ROC curves, which may be overly optimistic in such cases, Precision-Recall curves focus on the performance of the model with respect to the positive class, offering a clearer view of its ability to correctly identify positive instances while minimizing false positives. The area under the Precision-Recall curve provides a summary measure of the model’s performance, highlighting its effectiveness in handling the positive class.

A key observation of Precision-Recall curves is that Retrieving_FDT and Shifting_FDT exhibit better overall performance compared to the other two models. In particular, Retrieving_FDT maintained higher precision while recall increased, meaning it could identify true positive cases with fewer false positives, even as it became more sensitive. Shifting_FDT showed a similar trend, but with a slightly weaker balance between precision and recall. In contrast, Inhibiting_FDT and Decoding_FDT showed a sharper decline in precision as recall increased, indicating a higher number of false positives when these models aim for greater sensitivity. Decoding_FDT was the weakest performer, with precision dropping off significantly as the model attempted to capture more true positives, suggesting that this variable struggles to effectively distinguish true positives from false positives.

The overall model quality, summarized within bar charts in the figures, reinforces these observations. All of the FDT-based scores emerge as the most robust variables, achieving model quality scores ≥ 0.5 (Decoding_FDT (0.66), Retrieving_FDT (0.67), Inhibiting_ FDT (0.64), and Shifting_FDT (0.58)). Of the other variables, the ROCF_TimeCopy was the only one with a model quality score ≥ 0.50 (0.59), whereas the remaining showed small model quality scores: ROCF_Recall (0.32); ROCF_Copy (0.39); d2 attention scores (from 0.25 to 0.46), Symbol Search (0.27), Digit Span scores (from 0.29 to 0.33) and Zoo Map tests (0.33). These findings suggest that most neurocognitive variables have small predictive power, and that only mental inhibition scores could be used in clinical settings.

## 4. Discussion

This study revealed that although women with FMS exhibited deficits in different neurocognitive domains such as selective attention, long-term visual memory, processing speed, and mental inhibition compared with women without FMS, only the domains of mental inhibition exhibited moderate diagnostic accuracy to properly discriminate between women with and without FMS.

Attention has been a critical domain in studies of patients with FMS, with most research indicating that individuals with FMS experience deficits in attention at various levels [34]. This can be explained by the fact that attention and nociceptive pain processing share brainstem networks, including areas such as the medial and lateral prefrontal cortex as well as the anterior cingulate, which are all involved in both attention and nociceptive processing. Chronic pain may thus reduce attention capacity due to the greater demands on these shared brainstem areas [35]. It has been hypothesized that altered nociceptive pain processing, a key factor in the pathogenesis of FMS [36], may contribute to attention deficits, with higher levels of sensitization leading to greater attentional impairments. However, the current study did not find that attention deficits were a key factor in distinguishing between women with and without FMS [37,38].

Memory loss is another neurocognitive domain of clinical relevance in FMS, as memory deficits can affect patients’ ability to make decisions related to past events or situations [39]. These deficits can lead to feelings of inadequacy in daily life, potentially causing stress and anxiety. Further, memory impairments can hinder patient engagement, and the retention of information provided during therapy [39], which may affect treatment approaches requiring memory retention, e.g., neuroscience education [40]. Like attention deficits, changes in the brainstem, particularly within the hippocampus, which are associated with altered nociceptive processing [41,42], may explain memory loss in FMS patients. However, in our study, memory deficits were also not able to differentiate between women with and without FMS.

Mental inhibition, an important executive function, was also investigated in this study. The literature on mental inhibition deficits in this condition is mixed [43], with some studies not identifying any deficit [9], while others report moderate to large impairments in FMS patients [10]. A recent review concluded that mental inhibition was among the most significant deficits in FMS, suggesting it plays a key role in executive dysfunction [44]. As with other cognitive deficits, mental inhibition is influenced by the overlap between brainstem networks involved in nociceptive processing and cognitive functions, supporting the idea that chronic pain results in varied cognitive impairments [45]. The primary finding of the current study was that mental inhibition, as measured by the 5-Digit Test (FDT), was the only neurocognitive domain capable of distinguishing between women with and without FMS. Mental inhibition had the highest discriminatory power, with ROC values above 0.7, indicating a moderate ability to differentiate between the groups. However, specificity values were low, indicating that while FDT scores can help to identify the presence of mental inhibition deficits in women with FMS, the test by itself cannot reliably confirm the absence of such deficits in women without FMS. Other neurocognitive variables had ROC values below 0.7, demonstrating poor discriminatory power and limiting their clinical utility. This finding supports the “inhibition network” concept, which posits that although nociceptive and cognitive processes share similar brainstem areas, they are organized into distinct networks [46].

The study results must be interpreted considering its limitations. First, only women with FMS were included, so the diagnostic accuracy observed may not apply to men with this condition. Second, while patients taking psychoactive drugs or other medications that could affect cognition were excluded, the potential influence of past medication use on executive functions cannot be ruled out. Finally, we used a battery of neurocognitive tests, assessing only specific cognitive aspects. Future research should employ a broader range of neuropsychological tests to cover a wider array of neurocognitive components.

## 5. Conclusions

This study found that while women with FMS showed deficits in selective attention, long-term visual memory, processing speed, and mental inhibition, only mental inhibition, as measured by the 5-Digit Test (FDT), demonstrated moderate diagnostic accuracy in distinguishing between women with and without FMS. However, its specificity values were low, suggesting that although FDT can be useful for identifying the presence of mental inhibition deficit in women with FMS, they do not reliably exclude the possibility of its absence in women without FMS.

## Figures and Tables

**Table 1:** Neurocognitive variables and executive functions evaluated.

Cognitive Domains	Neuropsychological Tests	Outcomes	Method of Administration
Visuospatial Memory	Rey-Osterrieth Complex Figure (ROCF)	ROCF_Copy	Visual/Oral
		ROCF_Recall	
		ROCF_TimeCopy	
Selective Attention	D2 Test of Attention	D2_TR	Visual/Manual (Paper)
		D2_TA	
		D2_TOT	
		D2_CON	
		D2_VAR	
		D2_O	
		D2_C	
Processing Speed	Symbol Search (WAIS-IV)	Total Score	Visual/Manual (Paper)
Working Memory	Digit Span Forward (WAIS-IV)	Span of digits	Auditory/Oral
	Digit Span Backward (WAIS-IV)	Auditory Working Memory	
	Digit Span Sequencing (WAIS-IV)	Auditory Working Memory	
Planning/Decision	Zoo Map Test	Total Score	Visual/Manual (Paper)
Mental Inhibition	Five Digits Test FDT	Inhibiting_FDT	Visual/Oral
		Shifting_FDT	
		Decoding_FDT	
		Retrieving_FDT	

WAIS-IV: Wechsler Intelligence Scale for Adults-IV; d2_TR = total number of items answered; d2_TA = number of items answered correctly; d2_O = errors of omission committed; d2_C = commission errors made; d2_TOT = number of elements processed minus the total number of errors committed; d2_CON = number of relevant elements marked minus the number of commissions; d2_VAR = variation index; Inhibiting_FDT = time in seconds to read numeric items; Shifting_FDT time in seconds to read non-numeric items; Decoding_FDT = time in seconds to read all numeric items; Retrieving_FDT = time in seconds to read all non-numeric items.

**Table 2:** Means and standard deviations (SD) of neurocognitive and executive functions in women with and without fibromyalgia [13].

	Fibromyalgia (*n* = 129)	Controls (*n* = 111)	*p*
ROCF_Copy *	30.4 (0.6)	32.7 (0.7)	0.043
ROCF_Recall *	12.0 (0.8)	16.0 (0.9)	0.004
ROCF_TimeCopy	3.1 (1.1)	3.9 (1.2)	0.692
d2_TR *	337.1 (10.1)	379.5 (11.3)	0.019
d2_TA *	110.2 (4.5)	131.7 (5.0)	0.007
d2_O	31.9 (3.7)	29.5 (4.2)	0.709
d2_C *	6.5 (1.3)	1.8 (1.4)	0.042
d2_TOT *	295.6 (10.0)	345.6 (11.2)	0.005
d2_CON *	104.9 (4.9)	130.2 (5.5)	0.004
d2_VAR	15.6 (0.8)	14.6 (0.9)	0.486
Symbol Search *	26.4 (0.9)	30.9 (1.1)	0.008
DSF	7.9 (0.2)	7.9 (0.2)	0.821
DSB	6.7 (0.2)	7.3 (0.2)	0.145
DSS	7.1 (0.2)	7.5 (0.3)	0.455
Decoding_FDT *	25.3 (0.8)	20.1 (0.9)	0.001
Retrieving_FDT *	29.7 (1.4)	22.0 (1.5)	0.001
Inhibiting_FDT *	46.8 (2.2)	37.9 (2.5)	0.024
Shifting_FDT	60.6 (2.9)	50.4 (3.3)	0.053
Zoo Map test	11.1 (0.4)	12.1 (0.5)	0.172

ROCF_Copy refers to the direct score in the copy phase of the Rey-Osterrieth Complex Figure; ROCF_Recall is the direct score in the delayed recall phase of the same test; d2_TR represents the total number of items attempted; d2_TA indicates the number of items correctly answered; d2_O refers to omission errors; d2_C refers to commission errors; d2_TOT is the total number of processed elements minus all errors; d2_CON is the number of relevant elements marked minus commissions; d2_VAR represents the variation index. DSF, DSB, and DSS correspond to Digit Span Forward, Backward, and Sequencing, respectively. Decoding_FDT measures the time (in seconds) to read numeric items; Retrieving_FDT measures the time to read non-numeric items; Inhibiting_FDT reflects the time to read each numeric group; Shifting_FDT is the time taken to read mixed numeric items. Lastly, the Zoo Map Test measures the direct score in executing the planning task. * Statistically significant between-group differences.

**Table 3:** Discriminant capacity of neurocognitive variables and executive functions to differentiate women with and without FMS.

Variables	ROC Value	95% CI	Cut-Off Point	Significance	Sensitivity	Specificity	Youden Index	Positive LR	Negative LR
ROCF_Copy	0.457	0.384–0.529	43.5	0.241	0.015	0.000	0.016	NA	NA
ROCF_Recall	0.391	0.320–0.462	36.0	0.003	0.000	0.000	0.000	NA	NA
ROCF_TimeCopy	0.662	0.594–0.731	3.07	0.001	0.473	0.216	0.266	0.603	2.44
d2_TR	0.341	0.273–0.410	600.0	0.001	0.000	0.000	0.000	NA	NA
d2_TA	0.323	0.255–0.390	236.0	0.001	0.000	0.000	0.000	NA	NA
d2_O	0.512	0.439–0.585	16.5	0.753	0.558	0.468	0.090	1.05	0.944
d2_C	0.527	0.454–0.601	0.5	0.462	0.651	0.595	0.057	1.60	0.587
d2_TOT	0.314	0.247–0.381	561.0	0.001	0.000	0.000	0.000	NA	NA
d2_CON	0.331	0.263–0.399	235.0	0.001	0.000	0.000	0.000	NA	NA
d2_VAR	0.528	0.455–0.602	9.5	0.449	0.837	0.757	0.080	3.44	0.215
Symbol Search	0.341	0.272–0.411	54.0	0.001	0.000	0.000	0.000	NA	NA
DSF	0.396	0.325–0.467	2.5	0.004	1.000	0.991	0.009	111.1	0.000
DSB	0.396	0.325–0.467	15.0	0.004	0.000	0.000	0.000	NA	NA
DSS	0.361	0.291–0.431	0.5	0.001	1.000	0.991	0.009	111.1	0.000
Decoding_FDT	0.724	0.660–0.788	22.5	0.001	0.504	0.162	0.342	0.601	3.06
Retrieving_FDT	0.739	0.676–0.802	21.5	0.001	0.853	0.486	0.366	1.660	0.302
Inhibiting_FDT	0.708	0.642–0.773	40.5	0.001	0.566	0.225	0.341	0.730	1.929
Shifting_FDT	0.653	0.584–0.722	54.5	0.001	0.512	0.288	0.223	0.719	1.694
Zoo Map Test	0.401	0.330–0.473	17.0	0.007	0.000	0.000	0.000	NA	NA

## Data Availability

Materials and analysis code for this study are not available in any repository; however, we will make our data accessible upon request to the corresponding author.
